# Juvenile Amyotrophic Lateral Sclerosis: A Case Report of a Rare and Aggressive Presentation in a 22-Year-Old Filipino Male

**DOI:** 10.7759/cureus.68579

**Published:** 2024-09-03

**Authors:** Kimberly Po, Marietta Olaivar

**Affiliations:** 1 Section of Neurology, Department of Clinical Neurosciences, University of the East Ramon Magsaysay Memorial Medical Center, Quezon City, PHL; 2 Section of Neurology, Department of Internal Medicine, Cardinal Santos Medical Center, San Juan City, PHL

**Keywords:** : juvenile amyotrophic lateral sclerosis, motor neuron disease, filipino als, riluzole, case report

## Abstract

Amyotrophic Lateral Sclerosis (ALS) is a rare neurodegenerative disorder primarily affecting adults, but juvenile-onset ALS is exceptionally rare. We report a rare case of a 22-year-old Filipino male patient who exhibited early-onset weakness, muscle atrophy, and tongue fasciculations, followed by rapidly progressive dysphagia and respiratory distress. Electromyography - Nerve Conduction Velocity (EMG-NCV) findings showed evidence for a chronic, active predominantly motor neuronal-axonal loss type of neuropathy involving the tongue and limb muscles bilaterally consistent with a motor neuron disease. The patient was treated with riluzole with no significant improvement in symptoms. Despite multidisciplinary interventions, the disease rapidly progressed, highlighting the challenges in managing juvenile ALS cases. This case report emphasizes the importance of considering ALS in the differential diagnosis of progressive motor dysfunction in younger patients and the complexities involved in their care.

## Introduction

Juvenile Amyotrophic Lateral Sclerosis (Juvenile ALS) is an exceedingly rare neurodegenerative disorder characterized by the progressive degeneration of motor neurons in the spinal cord and brainstem, leading to severe disability and premature death [1]. Unlike adult-onset ALS, the juvenile variant manifests in individuals under the age of 25, posing unique diagnostic and therapeutic challenges due to its atypical presentation and limited data [2,3,4]. This case report presents the account of a young Filipino male diagnosed with juvenile ALS, highlighting its distinct clinical features, diagnostic considerations, and management challenges specific to this population. This case report aims to contribute valuable clinical insights, stimulate further research, and enhance awareness regarding juvenile ALS as the scarcity of information on juvenile ALS poses significant challenges for healthcare professionals, making it crucial to share and analyze individual cases to deepen our understanding of this rare condition.

## Case presentation

We present a case of a 22-year-old, male, Filipino, 3rd-year college student who came in with a gradually progressive 8-month history of difficulty in swallowing and speaking associated with excessive hypernasality of voice. Eight months prior to consult, the patient experienced easy fatigability during speaking, occasional aspiration episode on liquids, and hypernasal speech with a sensation of post-nasal drip. Despite these symptoms, he continued daily activities and reported no end-of-day fatigue. The subsequent months showed worsening of symptoms leading to seeking ENT evaluation with unremarkable video laryngoscopy findings hence was recommended for neurological consultation At the initial neurologic consultation, the patient was recommended to undergo Cranial and Cervical MRI, both of which returned unremarkable results. A follow-up consultation with a different neurologist, after reviewing the negative MRI findings, resulted in a prescription for coenzyme and vitamin B complex. Due to the consideration of a motor neuron disease, an Electromyography - Nerve Conduction Velocity (EMG-NCV) test was recommended; however, the procedure was postponed due to financial constraints. In the following months, his speech became increasingly hypernasal, and he experienced muscle stiffness, weakness in the right distal extremities, and neck pain causing him to seek a neurologic consult in our institution.

Prior to experiencing these symptoms, the patient did not have any medical problems, previous hospitalizations muscle pain, traumas, or adverse response to vaccinations. Additionally, there were no recent infections. Birth, maternal and developmental history were generally unremarkable. There was no history of any motor neuron disease in the family.

On examination, he had an intact mental status exam with noted hypernasality of speech, spastic dysarthria, and palatal tremors with tongue fasciculations. The muscle strength in the left lower limb was rated as 4+/5 for the proximal muscles and 4/5 for the distal muscles, with the overall strength of the left lower limb also being 4/5. Tendon reflexes were diminished in both the left lower and upper limbs but increased in the right lower and upper limbs. Sensory examination results were normal. Significant muscle atrophy was observed in the thenar, bulbar, and proximal deltoid muscles. Hoffman’s sign was present on the right upper extremity. 

The simultaneous presence of both upper motor neuron (UMN) and lower motor neuron (LMN) signs, along with progressive muscle weakness and atrophy, remains a distinctive feature of ALS, ruling out other probable differential diagnosis such as Guillain-Barré syndrome (GBS) and myasthenia gravis. Based on the El Escorial Criteria, the patient fits under the category of probable Juvenile ALS since UMN and LMN signs are present in two regions.

EMG-NCV findings showed evidence for a chronic, active predominantly motor neuronal-axonal loss type of neuropathy involving the tongue and limb muscles bilaterally consistent with a motor neuron disease (Table 1). The needle EMG study of the patient showed neuropathic change indicative of a chronic, active partial denervation process in distal and proximal muscles of the upper limbs as well as of the tongue muscle (genioglossus) on the right. In the lower limbs, chronic neuropathic process characterized mainly by motor unit remodeling seen in leg muscles sampled, left worse than right. No spontaneous activities were recorded.

**Table 1:** Needle EMG Examination R: right; L: left; Fibs: fibrillation potentials; Fasc: fasciculation potentials; PSW: positive sharp waves; MUPs: motor unit potentials

Muscle	Insertional Activity	Spontaneous Activity			Motor Unit Potentials
		Fibs	PSW	Fasc	
Tongue Muscle					
Genioglossus R	Increased	+1	None	None	Markedly reduced number of voluntarily activated motor unit potentials noted in right tongue muscle. Large motor units reaching up to 8mV seen.
Upper Limb					
Deltoid L	Increased	+1	+1	None	Mild to moderately reduced number of voluntarily activated motor unit potentials in left deltoid and both biceps: within normal in all other muscles sampled. Large motor units of increased duration some of which are polyphasic seen in left deltoid (5-6mV), both biceps (7-10mV), left flexor carpi radialis and left first dorsal interosseous (7-8mV). Most activated MUPs retain normal form, amplitude and duration.
Biceps Brachii L	Increased	+1	+2	None	
Biceps Brachii R	Increased	+1	+2	None	
Flexor Carpi Radialis L	Increased	None	+2	None	
Flexor Carpi Radialis R	Increased	None	+1	None	
Extensor Digitorum Communis L	Increased	None	+1	None	
First Dorsal Interosseous L	Normal	None	None	None	
Lower Limb					
Rectus Femoris L	Normal	None	None	None	Mild to moderately reduced number of voluntarily activated motor unit potentials in both gastrocnemius, left tibialis anterior and left rectus femoris; within normal in right tibialis anterior and rectus femoris. Large motor units of increased duration seen in both gastrocnemius (5-20mV). left tibialis anterior (5-6mV) and left rectus femoris (5mV). Most activated MUPs stil retain normal form, amplitude and duration.
Rectus Femoris R	Normal	None	None	None	
Tibialis Anterior L	Normal	None	None	None	
Tibialis Anterior R	Normal	None	None	None	
Gastrocnemius L	Normal	None	None	None	
Gastrocnemius R	Normal	None	None	None	

The patient was started with Riluzole failed to elicit to a treatment response and did not show any improvement in the symptoms. The patient eventually had marked progression of the bulbar symptoms and he eventually expired due to aspiration pneumonia.

## Discussion

Amyotrophic Lateral Sclerosis (ALS) is a neurodegenerative disorder typically onset in adulthood, marked by gradual muscle weakness and atrophy. While 10% of ALS cases are hereditary, and 90% are sporadic, most juvenile ALS cases have a genetic basis [1]. ALS is usually a progressive affectation of both upper and lower motor neurons. There is marked progressive muscle atrophy and diffuse paralysis would develop over time. The cause of death from ALS is due to respiratory failure and persistent aspiration episodes which occur within an average of 3-5 years from the onset of the disease [2]. The diagnosis is based on the standardized and most accepted diagnostic criteria for ALS - the El Escorial criteria which was developed by the World Federation of Neurology Research Group on Motor Neuron Diseases. Although these criteria were originally developed for research purposes, the levels of diagnosis depended on clinical assessment of the extent and distribution of UMN and LMN lesions (Table 2) [3].

**Table 2:** Revised El Escorial classification of ALS. ALS: amyotrophic lateral sclerosis; EMG: electromyography Source: Table 1, Revised El Escorial classification of ALS. Four anatomical regions - bulbar, cervical, thoracic, and lumbar - are included for disease stratification [5].

Diagnostic category	Inclusion criteria
Definite ALS	Presence of upper motor neuron and lower motor neuron signs in three anatomical regions
Probable ALS	Presence of upper motor neuron and lower motor neuron signs in at least two regions with upper motor neuron sign rostral to lower motor neuron signs
Probable ALS, laboratory results supported	Presence of upper motor neuron and lower motor neuron signs in one region with evidence by EMG of lower motor neuron involvement in another region
Possible ALS	Presence of upper motor neuron and lower motor neuron signs in one region or upper motor neuron signs in two or three regions, such as monomelic ALS, progressive bulbar palsy, and primary lateral sclerosis

Juvenile ALS is an uncommon form of ALS, typically linked to gene mutations like fused in sarcoma (FUS) and ALS2, with mutations in fused in sarcoma (FUS) as the most prevalent genetic cause [6]. Typically, jALS manifests with non-specific symptoms similar to adult-onset ALS such as limb weakness, fasciculations, and fatigue, which can include both upper/bulbar and lower motor neuron signs [7]. In a 2023 study by Raymond et al., which examined demographic characteristics of confirmed and likely juvenile ALS (jALS) cases in a large ALS patient cohort from the National ALS Registry between the years 2010 and 2018, jALS was found to be more common in non-white individuals (54.5%), and the condition was over three times more prevalent in males than females (79.5% vs. 20.5%) [8]. Moreover, the absence of dependable biomarkers to distinguish ALS from other types of motor neuron diseases (MND) creates significant challenges in diagnosis. The diagnosis of ALS can be difficult during the early stage since it shows great variability in presentation and a broad spectrum of disorders mimic the disease. Hence, diagnostics such as the EMG-NCV play a crucial role in arriving at the correct diagnosis. The cornerstone of managing amyotrophic lateral sclerosis (ALS) involves prompt and effective symptom management. Employing a multidisciplinary team approach is crucial for assisting patients in handling the ongoing neurological decline associated with the disease. Currently, there are no medications that provide significant clinical improvement for individuals with ALS [8]. This case report sheds light on the clinical and genetic features of juvenile ALS, emphasizing the importance of timely diagnosis and comprehensive supportive care. The challenges in managing juvenile ALS, including limited treatment options and the impact on the quality of life for both the patient and their family, are discussed. Management of motor neuron disease in the Philippines is largely inadequate and calls for the implementation of improvement in the health care system [9].

## Conclusions

Juvenile ALS remains a diagnostic and therapeutic challenge due to its rarity and aggressive course. Early recognition, multidisciplinary care, and ongoing research are essential in improving the prognosis and quality of life for affected individuals. This case underscores the urgent need for increased awareness, research, and support for patients and families grappling with this devastating condition.

From the patient's perspective, particularly in a third-world country with limited access to essential medications like riluzole, the burden of progressive disease at a young age is profoundly distressing. The mother’s experience highlights the immense emotional and financial strain faced by a lower socio-economic family managing such a diagnosis. The lack of access to necessary treatments and support services exacerbates their challenges, amplifying feelings of helplessness and frustration. This case underscores the urgent need for improved access to medications and support systems in under-resourced settings, as well as addressing the broader socio-economic factors that intensify the impact of progressive diseases.
